# *Toxoplasma gondii* infection in people with schizophrenia is related to higher hair glucocorticoid levels

**DOI:** 10.3389/fpsyt.2024.1286135

**Published:** 2024-02-16

**Authors:** Emy Beaumont, Jacques Brodeur, Frédéric Thomas, Antoine M. Dujon, and Consortium Signature, Sonia J. Lupien

**Affiliations:** ^1^ Institut Universitaire en Santé Mentale de Montréal, Center for Studies on Human Stress, Montréal, QC, Canada; ^2^ Research Center, Institut Universitaire en Santé Mentale de Montréal, Montréal, QC, Canada; ^3^ Department of Psychology, Université de Montréal, Montréal, QC, Canada; ^4^ Department of Biological Sciences, Université de Montréal, Montréal, QC, Canada; ^5^ Center for Ecological and Evolutionary Research on Cancer (CREEC), Université de Montpellier, Montpellier, France; ^6^ Center for Integrative Ecology, School of Life and Environmental Sciences, Deakin University, Waurn Ponds, VIC, Australia; ^7^ Depatment of Psychiatry and Addiction, Université de Montréal, Montréal, QC, Canada

**Keywords:** parasite, mental health, cortisol, infection, toxoplasmosis, host manipulation

## Abstract

**Introduction:**

*Toxoplasma gondii* (TG) is a common protozoan parasite infecting approximately one third of the human population. Animal studies have shown that this parasite can manipulate its host behavior. Based on this, human studies have assessed if TG can be involved in mental health disorders associated with important behavioral modifications such as schizophrenia. However, results have been discrepant. Given that TG has a strong impact on fear and risk-taking processes in animal studies and that fear and risk-taking behaviors are associated with the human stress response, we tested whether glucocorticoid biomarkers (salivary and hair) differ in people with schizophrenia and controls as a function of TG status.

**Methods:**

We measured TG antibodies in blood samples, as well as salivary and hair glucocorticoid levels in 226 people with schizophrenia (19.9% women, mean age = 39 years old) and 129 healthy individuals (controls) (45.7% women, mean age = 41 years old).

**Results:**

The results showed that people with schizophrenia infected with TG presented significantly higher hair glucocorticoid concentrations than non-infected people with schizophrenia. This effect was not found in control participants. No effect was observed for salivary glucocorticoid levels. Additionally, there were no associations between TG infection and positive psychotic symptoms nor impulsivity.

**Discussion:**

These results show that people with schizophrenia present high levels of hair glucocorticoid levels only when they are infected with TG. Further studies performed in populations suffering from other mental health disorders are needed to determine if this effect is specific to schizophrenia, or whether it is generalized across mental health disorders.

## 1 Introduction

Parasitism refers to a close relationship between two organisms, wherein one organism, known as the parasite, relies on the other organism, known as the host, to obtain certain benefits (usually food; Rohde, 2013). Many parasites follow a direct life cycle, meaning they use a single host throughout their life. Alternatively, some parasites adopt an indirect life cycle by exploiting a definitive host, as well as one or more intermediate hosts. The parasite cannot reach sexual maturity and complete its life cycle until it has reached its definitive host (1). In many cases, a parasitized animal does not behave in the same way as a healthy animal (2). In the 80s, the evolutionary biologist Richard Dawkins proposed a concept called the “extended phenotype”, that can explain this phenomenon (3). It refers to the idea that the genes of an organism can have effects that extend beyond their physical body and behavior and have consequences on their environment, potentially influencing other organisms in the process. In the case of parasites, some of them have evolved to control the behavior of their host, enhancing their own survival and the reproductive success of their genes to the next generation (4).

*Toxoplasma gondii* (TG), an intracellular protozoan parasite that can only complete its life cycle when infecting Felidae family members, is known to manipulate the behavior of its intermediate host (4–6). One of the most interesting discoveries about TG is that when it infects mice and rats, this results in a loss of fear towards felines, favoring the parasite transmission to its definitive host (7, 8). Usually, the rodents’ innate aversion to feline odors keeps them away from their predator, diminishing their probability of getting eaten. However, when infected by TG, this aversion turns into attraction, which leads to more risky behaviors from the rodent in front of the predator, a process called the “fatal attraction phenomenon” (7). A new study suggests that this loss of fear is not specific to feline predators. In fact, TG’s effects on mice can be more generic, making them more eager to explore and be less fearful of other species too (9). Studies have also reported some differences in responses to feline odors in chimpanzees, and even in humans (6, 10).

Threatening situations, for example when a mouse encounters a predator such as a cat, normally activate the fear system that leads to a physiological stress response. When the brain detects a threat, the amygdala activates the hypothalamic-pituitary-adrenal (HPA) axis. This axis ultimately leads to the production of glucocorticoids (11, 12). Hence, the lack of fear in TG infected rats may result from a diminished activation of the amygdala, leading to an inhibited stress response and lower circulating levels of glucocorticoids. This is supported by a study conducted on rats, some of which were experimentally infected with TG (13). Results showed that infected rats presented lower levels of circulating glucocorticoids.

However, in one study, Fallah et al. compared glucocorticoid levels in rats injected with TG and controls (14). The results showed that 14 days after infection, TG infection was associated with increased glucocorticoid levels. This is consistent with the results of a meta-analysis that showed that parasitic infection tends to increase glucocorticoid levels (15). Moreover, TG infected rats visited more often the open arms of an elevated maze, revealing decreased anxiety levels in TG infected animals. Presence of low anxiety behaviors in association with high glucocorticoids levels can be explained by risk-taking behaviors. It is thus possible that because they are less anxious, TG infected animals present more risk-taking behaviors, which increases activation of the HPA axis and leads to higher circulating levels of glucocorticoids.

Humans are one of the intermediate hosts of TG, meaning that similarly to animals, they can get infected by the parasite. *Toxoplasma gondii* is thought to infect approximately one third of the world population, with variations in prevalence observed across geographical regions, as well as among different age groups and sex (16, 17). Once it has infected a human, TG can induce latent infection by establishing permanent cysts in different locations, including the brain (18). Infection can be detected by the presence of TG-specific antibodies in serum samples. The most commonly used antibody types are immunoglobulin G and M (IgG and IgM). The concentration of IgG antibodies remains high for life following a TG infection, while the concentrations of IgM antibodies indicate a more recent infection since these antibodies can only be detected for a little more than a year (19).

TG has also been shown to alter human behavior. Although the relationship between TG and fear in humans remains unclear, some studies have suggested associations between TG infection and modified fear responses and/or behavioral shifts towards risk-taking in humans. For example, TG infection in humans has been shown to be associated with increased aggression and impulsivity (20–22). This parasite has also been associated with more road accidents, which might be explained by poor impulse regulation and higher risk-taking behaviors (23, 24). A study also found a link between TG and entrepreneurial activity, with infected individuals being more likely to have started their own business and expressing a lower fear of failure (25).

Since behavioral changes are apparent in many mental health disorders, other studies have assessed whether TG infection is more frequent in some psychiatric disorders compared to controls. Schizophrenia, a disorder associated with important behavioral changes, has frequently been tested for its association with TG (26). This mental health disorder is characterized by positive symptoms such as delusions and hallucinations, disorganized speech and behavior and/or negative symptoms such as restricted emotional expression or motivation (27). Higher self-report impulsivity is observed in people with schizophrenia, but risk-taking behaviors vary across them. Some show risk aversion while others present increased risk-taking behaviors (28, 29).

So far, studies have shown that schizophrenia is the mental health disorder with the strongest and most established association with TG, with many studies showing that a greater percentage of people with schizophrenia are infected with the parasite compared to control populations (odds ratio between 1.81 and 2.73) (18, 26, 30–32). The mechanism explaining greater TG infection rates in people with a diagnosis of schizophrenia is unknown. This could be the result of some characteristics of people with schizophrenia (for example, greater cat ownership during life (33)) making them more at risk of getting infected. It could also be explained by a higher risk of developing schizophrenia in infected individuals. But if it is the case, considering that not all TG infected individuals will develop schizophrenia, some unknown factor(s) must come into play. Given the effects of TG on risk-taking behaviors and self-induced stress reactivity in animal studies, it is possible that TG infected people with schizophrenia would present more changes in glucocorticoid levels and greater risk-taking/impulsivity behaviors compared to controls.

To test if TG could be linked to differences in the stress responses, Shirbazou et al. recruited 180 patients (regardless of their diagnosis) in a hospital and tested them for the presence of TG IgG antibodies (34). They also measured their blood glucocorticoid concentrations (cortisol), as well as self-reported depression, anxiety and stress. Their results showed that infection to TG was related to higher glucocorticoid concentrations and more subjective stress. A few years later, Abdelazeem et al. recruited 150 patients in a military hospital, 100 of which were infected with TG (35). In their study, blood glucocorticoid levels were also higher in TG seropositive individuals. These results show that TG infection can be associated with higher glucocorticoid levels in humans. However, it is important to note that glucocorticoid measure was taken with single blood samples from each participant in these studies, which means that the measure could have been affected by many other contextual factors.

Only one study compared glucocorticoid levels in TG infected and non-infected people with schizophrenia and it reported no difference between groups (36). However, it was not clear whether blood samples used to assess circulating levels of glucocorticoids were obtained at the same time of the day and if not, whether statistical analyses controlled for this. Glucocorticoid concentrations fluctuate according to a circadian rhythm with highest levels in the morning period and declining levels over the day (37). Also, blood sample analyses measure both the bound and unbound portions of glucocorticoids which can be problematic. Indeed, only the unbound portion of the steroid can cross the blood-brain barrier and affect behavior. Consequently, the focus should be on unbound steroids when examining associations between glucocorticoids and behavior. In contrast to blood, samples of saliva allow for the assessment of the unbound portion of the hormone, and it reflects the circulating levels of the hormones at the time of sampling (12, 38, 39). However, this measure is strongly affected by the context at the moment of sampling, and may poorly reflect normal, long-term glucocorticoid secretion (40). A measure of glucocorticoids secreted in the long-term can be obtained through hair samples, providing an indication of chronic stress. Glucocorticoid levels accumulate in hair samples and because average hair growth rate is about 1 cm/month, 3cm of hair from the scalp constitute a measure of glucocorticoids produced in the last three months (40).

The first objective of this study was to test whether two unbound glucocorticoid biomarkers (salivary and hair) differ in people with schizophrenia and controls as a function of TG status. The second objective was to test whether TG infection is associated with specific psychotic symptoms, and to test the role of impulsive behaviors. As current knowledge on the subject is very limited, no hypothesis was defined as to the directionality of effects.

## 2 Material and methods

### 2.1 Study population

Data for this study comes from the Signature Biobank project (https://www.banquesignature.ca) that was developed in 2012 by the Institut Universitaire en Santé Mentale de Montréal (IUSMM), a psychiatric institution in the province of Québec, Canada. This large-scale biobank aims to accumulate biological samples, as well as psychiatric and psychosocial data from patients using the IUSMM’s psychiatric services and control participants. All patients were invited to take part in this project. This biobank includes, among other things, psychiatric diagnoses made by the treating psychiatrists of the hospital at the time of their admission to the psychiatric emergency, serum, saliva and hair samples, and questionnaires assessing psychotic symptoms and impulsivity. Data collection (biological samples and psychological measures) was done upon patients’ admission to the hospital. Control participants of the Signature Biobank were not recruited in the hospital. Rather, they were recruited with an ad and went through a screening questionnaire by a phone interview. Among other things, this interview aimed to exclude participants that had been admitted in a psychiatric emergency in the last 5 years. The full screening questionnaire is available in the Supplementary Material.

For the present study, participants from the Signature Biobank were selected on the basis of having a schizophrenia diagnosis or being part of the control group and having available biospecimen to measure TG infection (blood samples) and glucocorticoid levels (saliva and/or hair samples). Data from 226 people with a diagnosis of schizophrenia (19.9% women) and 129 healthy controls (45.7% women) were used in this study. Comparisons were done using chi-squares (sex and education level), a t-test (age) and a Mann-Whitney U test (personal income) to compare the two groups (schizophrenia and control) on sociodemographic variables. Descriptive analysis of the sample is presented in **Table 1**. The number of hair samples available was lower, with 96 samples for people with schizophrenia (28.1% women) and 95 for controls (56.8% women).

**Table 1:** Description of the sample.

Measure	Control	Schizophrenia	*p*-value
Sex			
n	129	226	
Women	59 (45.7%)	45 (19.9%)*	<.001
Men	70 (54.3%)	181 (80.1%)*	
Age (years)			
n	129	226	
Mean	41.0	39.1	.267
SD^1^	16.4	13.5	
Minimum	18	18	
Maximum	82	77	
Personal income ($)			
n	129	219	
Median	27,000$	12,000$	<.001
IQR^2^	39,500$	3,500$	
Minimum	0$	0$	
Maximum	250,000$	50,000$	
Education level			
n	128	205	
No SSD^3^	8 (6.3%)	87 (42.4%)*	<.001
SSD	17 (13.3%)	60 (29.3%)*	
Post-Secondary	25 (19.5%)	40 (19.5%)	
University degree	78 (60.9%)	18 (8.8%)*	

^1^SD: Standard deviation.

^2^IQR: Interquartile range.

^3^SSD: Secondary School Diploma.

*p<0.05.

### 2.2 Disclosures

This study was approved by the Research Ethics Board of the Center intégré universitaire de santé et de services sociaux de l’Est-de-l’Île-de-Montréal, Québec, Canada, in August 2022. This project was preregistered prior to the analyses on the Open Science Framework platform on November 9^th^, 2022 (https://osf.io/3qhft/). To keep the analyses as pertinent and concise as possible, some analysis changes were done afterward, but the goal remained the same: testing whether TG infection in people with schizophrenia or healthy individuals is associated with differences in glucocorticoid levels.

### 2.3 Measures

#### 2.3.1 *Toxoplasma gondii* antibodies IgG and IgM

Serum samples were obtained by centrifugation of blood samples, and frozen until tested for the presence of TG antibodies. Samples were first tested for IgG antibodies to TG using Enzyme-Linked Immunosorbent Assay (ELISA) kits from DRG International (Springfield, NJ, USA) following manufacturer’s instructions. Results were obtained based on the optical density (OD) of the samples compared to the OD of a standard, which served as cut-off (ODCO). All samples were tested in duplicates, and the means were used to determine seropositivity. Samples with an OD > ODCO + 10% were considered positive while those with an OD < ODCO - 10% were considered negative. Samples with an OD in between those values (considered a grey zone) were retested. Given the small probability of being positive for IgM antibodies to TG while being negative for IgG antibodies to TG, only the IgG positive samples were tested for IgM. Samples were tested for IgM antibodies to TG using the same procedure.

#### 2.3.2 Salivary glucocorticoid

Saliva samples were obtained from the participants upon their admission to the psychiatric emergency (for the schizophrenia group) or the visit to the Signature Center (for the control group), and then frozen at -80°C until tested. Timing of saliva sample collection varied among participants, but the sampling times were noted and controlled for in the analyses of results. Samples were used to determine the concentrations of unbound glucocorticoid using Salimetrics’ Salivary Cortisol Enzyme Immunoassay kits. All samples were assayed in duplicates, and the mean concentration of both analyses was used. Considering that saliva samples were taken at participants’ admission to the hospital (for the schizophrenia group), they assess a hormonal response to an unusual situation and are not necessarily representative of their normal daily stress levels.

#### 2.3.3 Hair glucocorticoid

Hair samples were also obtained upon admission to the hospital or the visit to the Signature Center and were first kept in a dark and dry environment at ambient temperature. Hair strands were cut from the posterior vertex position as close as possible to the scalp. Samples were measured, cut and weighed. Hair samples measuring 3cm, starting from the root, were prepared in order to measure 3 months of glucocorticoid concentrations. Glucocorticoid extraction was performed based on the laboratory protocol described by Kirschbaum et al., using IBL International Luminescence Immunoassay kits (41). This hair glucocorticoid measure, representing the glucocorticoid produced in the last 3 months, is more representative of long-term hormone secretion and does not fluctuate with the time of sampling. All sample were assayed in duplicates, and the mean of both results was used in this study.

#### 2.3.4 Psychotic symptoms

Psychotic symptoms were measured by the Psychosis Screening Questionnaire (PSQ; (42); French translation: (43)). The PSQ measures the presence or absence of 5 psychotic symptoms (i.e., hypomania, thought interference, persecution, perceptual abnormalities, strange experiences) in the last 12 months using a total of 12 questions answered by “yes”, “no” or “maybe”. The respondent answers between 5 and 12 questions depending on their responses. A global score can also be calculated, representing the respondent’s number of psychotic symptoms (on a total of 5). This questionnaire has good specificity (95%) and sensitivity (97%) (42).

#### 2.3.5 Impulsive behavior

Five factors that can lead to impulsive behavior were assessed using the French UPPS-P Impulsive Behavior Scale, i.e., negative urgency, positive urgency, lack of premeditation, lack of perseverance, and sensation seeking (44). This 20-item questionnaire was developed based on the UPPS impulsive behavior scale (45). It contains 4 questions assessing each of the 5 sub-scales. Items are scored on a Likert scale from 1 (“I agree strongly”) to 4 (“I disagree strongly”). This questionnaire has good internal reliability coefficients (Cronbach’s α between.70 and.84) and test-retest stability indices between .84 and .92 (44).

### 2.4 Analyses

#### 2.4.1 Preliminary analyses

Analyses were conducted using IBM SPSS Statistics 28. There were 7 missing data for the variables of psychotic symptoms and 5 for the impulsive behavior measures. Salivary and hair glucocorticoid data were log transformed as their distributions were asymmetrical (skewness of 9.31 and 8.75, respectively) and heavy-tailed (kurtosis of 120.70 and 86.99, respectively). After this transformation, salivary and hair glucocorticoid skewness was -0.08 and 1.51, respectively, and kurtosis 1.88 and 3.99, respectively. However, salivary and hair glucocorticoid data are also presented in the table as non-log transformed values to facilitate comparison across studies. Given that only 5 participants in total (2 controls and 3 people with schizophrenia) tested positive for IgM antibodies to TG, we were unable to conduct the planned analyses with this variable due to lack of statistical power. We only could compare TG IgM prevalence between the groups.

Frequencies for each group on the studied variables were analyzed. Chi-squared tests were conducted to compare frequency of TG seropositivity (IgG and IgM) amongst people with schizophrenia and control participants in our sample. A logistic regression was also done to test the association between the group and TG IgG while controlling for the covariates age, sex, personal income, and education level. T-tests were also used to test the differences between controls and people with schizophrenia on the glucocorticoid variables of this study.

#### 2.4.2 Main analyses

To test whether glucocorticoid levels differed by TG status and/or group, two two-way ANCOVAs were performed. The type I error was set at 5% (α<0.05). Salivary glucocorticoid was the dependent variable (DV) in the first analysis. The TG status (presence or absence of IgG antibodies), the group (schizophrenia or control), and their interactions were included as the independent variables (IVs). Age, sex, personal income, education level, and the time of the saliva sample collection (to control for variations due to the circadian cycle) were included as covariates. Hair glucocorticoid was the DV in the second analysis. IVs were the same as the first analysis, and age, sex, personal income, and education level were included as covariates.

#### 2.4.3 Secondary analyses

To test whether psychotic symptoms and/or the number of psychotic symptoms were associated with TG, logistic regressions were performed for every sub-scale of the PSQ (i.e., hypomania, thought interference, persecution, perceptual abnormalities, and strange experiences; DVs) to verify the presence of an association between TG IgG antibodies and those psychotic symptoms. A linear regression was performed to verify the presence of an association between TG IgG antibodies and the global score of the PSQ. In those analyses, TG IgG was the IV and covariates were the same as before (sex, age, personal income, and education level).

Finally, to test if the link between TG and log hair glucocorticoid differed as a function of impulsivity, ANCOVAs were performed for each sub-scale of the French UPPS-P Impulsive Behavior Scale. Log hair glucocorticoids was included as the DV and the IVs were TG IgG, the impulsivity sub-scale score and their interaction. The covariates were also sex, age, personal income, and education level. Schizophrenia and control groups were tested separately.

Because of the number of analyses and covariates, and to prevent from false positive results, the type I error was set at 1% (α<0.01) for the secondary analyses.

## 3 Results

### 3.1 Preliminary analyses

When we compared people with schizophrenia and controls on TG infection, we found no group differences in IgG antibodies (χ^2^ (1, N = 355) = 0.28, *p* = 0.596) or IgM antibodies (χ^2^ (1, N = 59) = 0.09, *p* = 0.763). We also tested the association between the group and TG IgG with sex, age, personal income and education level as covariates using a logistic regression, and the result remained nonsignificant (OR = 1.08, CI_95 = _0.45-2.58, *p* = 0.859). Log salivary glucocorticoid concentrations were significantly higher in people with schizophrenia compared to controls (t(350) = 3.16, *p* = 0.002), but not the log hair glucocorticoid concentrations (t(189) = 1.85, *p* = 0.066), even though the differences were in the same direction as salivary glucocorticoids. Results are presented in **Table 2**.

**Table 2:** Comparisons of controls vs. people with schizophrenia on the variables used in this study.

Measure	Controls	Schizophrenia	*p*-value
Toxo IgG			
n	129	226	
Positive	20 (15.5%)	40 (17.7%)	.596
Negative	109 (84.5%)	186 (82.3%)	
Toxo IgM			
n	20	39	
Positive	2 (10.0%)	3 (7.7%)	.763
Negative	18 (90.0%)	36 (92.3%)	
Salivary glucocorticoid			
n	128	224	
Raw salivary glucocorticoid (μg/dl) M (SD)	0.33 (0.59)	0.38 (0.32)	
Log salivary glucocorticoid (log-μg/dl) M (SD)	-1.47 (0.73)	-1.22 (0.70)	.002
Hair glucocorticoid			
n	95	96	
Raw hair glucocorticoid (pg/mg) M (SD)	30.76 (74.43)	49.69 (161.66)	
Log hair glucocorticoid (log-pg/mg) M (SD)	2.82 (0.86)	3.07 (0.96)	.066

M= Mean.

SD= Standard deviation.

### 3.2 Main analyses

**Figure 1:**
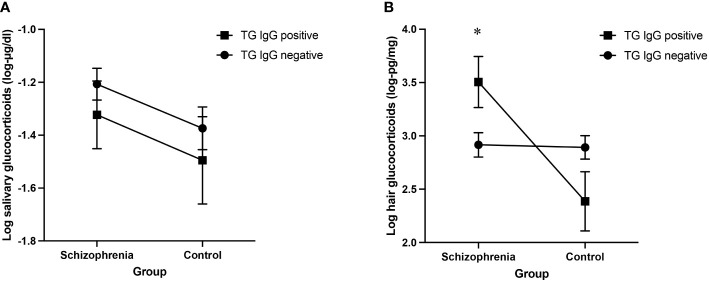
**(A)** Log salivary glucocorticoid adjusted means for TG infected and non-infected people with schizophrenia and control participants **(B)** Log hair glucocorticoid adjusted means for TG infected and non-infected people with schizophrenia and control participants. *R*esults of the ANCOVA analyses including age, sex, personal income, education level and time of saliva sample (1A) and age, sex, personal income and education level (1B) as covariates, error bars representing standard errors. *p<0.05.

We found no effect of TG IgG antibodies on log salivary glucocorticoid levels, F(1, 315) = 1.12, *p* = 0.290, and no interaction between TG and group, F(1, 315) = 0.00, *p* = 0.982. Results are presented in **Table 3A** and in **Figure 1A**.

**Table 3A:** Results of ANCOVA #1 comparing the log salivary glucocorticoid means of TG infected and non-infected people with schizophrenia and control participants, when adjusting for age, sex, personal income, education level, and time of saliva sample.

Variable	F	*p*-value	Partial η^2^
Group (schizophrenia/control)	1.659	.199	.005
TG IgG (presence/absence)	1.124	.290	.004
Group * TG IgG	0.001	.982	.000
Age	0.611	.435	.002
Sex	0.506	.477	.002
Time of saliva sample	22.637	<.001	.067
Personal income	0.629	.428	.002
Education level: SSD^1^	0.100	.752	.000
Education level: Post-secondary	1.513	.220	.005
Education level: University degree	0.935	.334	.003

^1^SSD: Secondary School Diploma.

However, we found a significant interaction between TG and group on log hair glucocorticoid levels, F(1, 175) = 8.38, *p* = 0.004, η_p_^2 = ^0.046. The partial eta squared (SS-effect/SS-total) of 0.046 indicates a small to moderate effect size, explaining approximately 4.6% of the variance of hair glucocorticoid levels in this analysis while adjusting for the other variables (46, 47). *Post-hoc* analyses revealed a significant simple effect of TG on hair glucocorticoid concentrations only in people with schizophrenia, F(1, 175) = 5.50, *p* = 0.020, η_p_^2 = ^0.030. Results are presented in **Table 3B** and in **Figure 1B**.

**Table 3B:** Results of ANCOVA #2 comparing the log hair glucocorticoid means of TG infected and non-infected people with schizophrenia and control participants, with the potential confounders age, sex, personal income, and education level.

Variable	F	*p*-value	Partial η^2^
Group (schizophrenia/control)	6.679	.011	.037
TG IgG (presence/absence)	0.046	.831	.000
Group * TG IgG	8.377	.004	.046
Age	0.511	.475	.003
Sex	1.018	.314	.006
Personal income	1.075	.301	.006
Education level: SSD^1^	2.247	.136	.013
Education level: Post-secondary	0.325	.569	.002
Education level: University degree	0.039	.843	.000

^1^SSD: Secondary School Diploma.

### 3.3 Secondary analyses

As presented in **Table 4**, none of the psychotic symptoms were significantly associated with TG IgG. Therefore, in accordance with the preregistered plan, this path was not explored further.

**Table 4:** Logistic and linear associations between TG IgG and the PSQ measures.

PQS sub-scales (DV)	*OR*	*95% CI*	*p*-value
Hypomania	7.871	0.30-206.36	.216
Thought interference	1.174	0.50-2.73	.709
Persecution	0.776	0.32-1.88	.575
Strange experiences	0.714	0.32-1.58	.405
Perceptual abnormalities	0.756	0.33-1.73	.509
	β	t	*p*-value
Global score	-0.041	-0.752	.453

Results are presented for the IV TG IgG, while controlling for covariates age, sex, personal income and education level.

For impulsivity, we found no interaction effect of TG and impulsivity measures neither in people with schizophrenia nor in controls. Results are presented in **Table 5**.

**Table 5:** ANCOVA results of the interaction factor between TG IgG and the impulsivity measures for people with schizophrenia and control participants.

Impulsivity sub-scales * TG IgG	Schizophrenia			Control		
	F	*p*-values	Partial η^2^	F	*p*-values	Partial η^2^
Negative urgency	0.893	.528	.102	2.248	.072	.112
Positive urgency	0.435	.853	.039	2.396	.046	.148
Lack of premeditation	1.254	.287	.116	1.118	.359	.071
Lack of perseverance	0.134	.984	.010	1.196	.320	.061
Sensation seeking	1.207	.310	.133	0.965	.455	.077

Results are presented for the interaction of TG IgG and impulsivity measures (IVs) on log hair glucocorticoids (VD), while controlling for covariates age, sex, personal income and education level.

## 4 Discussion

The goal of this study was to determine whether glucocorticoid biomarkers (salivary and hair) differ in people with a diagnosis of schizophrenia and controls as a function of TG status. Potential associations between TG and psychotic symptoms and the role of impulsivity were also examined. 

First, we found that people with schizophrenia did not have TG infection in a greater proportion than controls. This result goes against the conclusions of many studies and meta-analyses published in the literature (26, 30–32). However, authors of two of those meta-analyses found evidence for a publication bias in favor of positive results, suggesting that the higher prevalence of TG infection in people with schizophrenia could be weaker than previously estimated (30, 31). It is thus possible that the absence of a relationship between TG status and group (schizophrenia/control) in this study is in line with studies suffering from the drawer effect in which negative results are not published.

Analyses also showed that salivary glucocorticoid levels were significantly higher in people with schizophrenia compared to controls. Disrupted HPA axis activity has been linked to many mental health disorders, including schizophrenia (48). This result is consistent with those of many other studies showing alterations of the HPA axis in some people with schizophrenia (48, 49). Elevated hair glucocorticoid levels have also been reported in various mental health disorders (50). But because hair analysis as a measure of HPA activity is relatively recent, few studies have been done assessing hair glucocorticoids in people with schizophrenia and results are heterogenous (51–54). Our results suggest that hair glucocorticoid levels do not differ in people with schizophrenia in comparison to controls.

Regarding the salivary and hair glucocorticoid levels as a function of TG status, we found no difference in people with schizophrenia and in control participants for salivary glucocorticoid levels, but hair glucocorticoid levels differed by TG status only in people with schizophrenia. Those infected with TG presented significantly higher levels of hair glucocorticoids compared to those not infected with TG.

Saliva samples were taken at different times of the day in a psychiatry emergency service where patients had arrived in crisis. It is thus possible that the context in which saliva samples were obtained explains the lack of effect on this biomarker. It is also possible that TG infection does not impact acute levels of glucocorticoids although differences emerge when we assess the long-term production of this stress hormone. Accordingly, we found greater concentrations of glucocorticoids in 3cm of hair samples, which represents 3 months of glucocorticoid production in people with schizophrenia infected with TG.

These results are very interesting because they go along with the animal study by Fallah et al. showing that TG infection is associated with increased levels of glucocorticoids. Apart from the possible increased risk-taking, a possible mechanism for the higher concentrations of hair glucocorticoids found in people with schizophrenia infected with TG lies in the immune system activity. Indeed, high levels of inflammation are often found in this population (55). Considering that the production of certain pro-inflammatory cytokines can lead to the secretion of glucocorticoids, it is possible that this greater immune activity, combined with the body’s immune response caused by TG infection, could lead to greater glucocorticoid production in the long term (56). However, the cross-sectional nature of this study does not allow to determine the directionality of the effect. Although it is possible that elevated hair glucocorticoid levels are a result of TG infection in people with schizophrenia, increased glucocorticoid levels could also be a factor that increases vulnerability to schizophrenia in TG infected individuals. This goes in line with studies showing that the HPA axis can be altered in individuals with schizophrenia (48, 49, 52). Some studies also showed that glucocorticoids can inhibit the animal’s immune response to TG, facilitating its replication (57–59). This could lead to an increased risk of developing schizophrenia in TG infected individuals with high hair glucocorticoid levels. Another possibility is that glucocorticoids produced by an individual at the time of TG infection amplify its effect on the brain. Studies have shown that activation of the HPA axis can lead to an increase in blood-brain barrier permeability (60). As mentioned previously, TG manages to lodge permanently in the human brain cells. Although the mechanism by which it does so is unknown, some have been proposed, such as growth across endothelium, direct transmigration and trafficking within parasitized leukocytes (61). It is possible that stress facilitates the access of the parasite to the brain, increasing vulnerability to schizophrenia.

No association was found between TG and the different psychotic symptoms, or with the number of symptoms participants had. This suggests that TG could be linked to schizophrenia disorder without specific associations with some symptoms. It is important to take into consideration that only psychotic symptoms were assessed, which does not consider the extent of schizophrenic symptomatology (e.g., negative symptoms). This questionnaire is also a self-reported measure and only determines the presence/absence of a symptom, without assessing the severity, which constitutes a limit. An additional assessment of schizophrenia symptoms from a healthcare worker or a family member, for example, could improve the reliability of the measure. Importantly, the medication taken by participants of the schizophrenia group before their admission to the hospital – an information we do not have – could have influenced their symptomatology at that time. Some studies suggest that some antipsychotic drugs could have anti-toxoplasmic activity, which could also influence the associations between TG and psychological measures such as the psychotic symptoms (62, 63).

In animals, TG infection is related to high levels of glucocorticoids and to risk-taking behavior and lack of fear (7–9, 14). In the present study, we assessed impulsivity as a proxy measure of risk-taking behaviors in humans. If TG-infected individuals with schizophrenia tend to put themselves in risky situations, this could lead to more frequent stress responses and explain why they produced more glucocorticoids in the last 3 months compared to TG non-infected people with schizophrenia. However, results showed that the association between the TG infection and log hair glucocorticoid levels in the schizophrenia group did not differ as a function of impulsivity measures. This suggests that the higher hair glucocorticoid levels found in TG-infected people with schizophrenia are linked to something other than higher risk-taking behaviors. However, it is important to take into consideration that we did not use a direct measure of risk-taking behavior, which is what animal studies do. What we consider a proxy, impulsivity, was assessed through a self-report measure. This method is based on the participant’s conscious evaluation of their own traits and is thus prone to many biases. A behavioral measure as the ones used in animal studies could be a good complement for the assessment of risk-taking behaviors.

This study is the first to show that hair glucocorticoid levels differ as a function of TG presence in people with schizophrenia. Our results suggest that HPA activity could be affected by TG in people with schizophrenia, a hypothesis that should be tested in future studies. A longitudinal study, for instance, could test whether increased hair glucocorticoids occur as a result of TG infection or if higher hair glucocorticoid levels before the infection predicts later development of schizophrenia. Interactions with other factors known to be linked to schizophrenia should also be explored. For example, it is now known that genetics play a role in schizophrenia (64). It is thus possible that some genes could increase the vulnerability to schizophrenia in TG infected individuals. Moreover, some genes also play a role in the HPA axis activity, which may explain differences in TG-glucocorticoid associations between individuals (65). More studies are needed to better understand how stress is related to TG and schizophrenia, but our results indicate that this path is worth exploring further.

In conclusion, results from this study show that TG infection is related to higher hair glucocorticoid levels in people with schizophrenia, but not in healthy controls, whereas salivary glucocorticoids do not seem to be affected by the parasite. This suggests that TG infection is linked to differences in long-term glucocorticoid secretion in people with schizophrenia, but not in short-term glucocorticoid secretion. These results highlight the relevance of exploring the role of stress in studying TG and schizophrenia.

## Data availability statement

Since data from this study stems from a large-scale joint project (Signature Project), data distribution must be done in accordance with the established data sharing policy. This implies that it cannot be openly shared, but those interested in using the data are welcome to make a request to the Signature biobank coordinator at signature.iusmm@ssss.gouv.qc.ca.

## Ethics statement

The studies involving humans were approved by the CÉR évaluateur du Center intégré universitaire de santé et de services sociaux de l’Est-de-l’Île-de-Montréal (CEMTL) - installation Institut universitaire en santé mentale de Montréal. The studies were conducted in accordance with the local legislation and institutional requirements. The participants provided their written informed consent to participate in this study.

## Author contributions

EB: Conceptualization, Data curation, Formal analysis, Investigation, Methodology, Project administration, Visualization, Writing – original draft, Writing – review & editing. JB: Investigation, Resources, Validation, Writing – review & editing. FT: Funding acquisition, Investigation, Validation, Writing – review & editing. AD: Validation, Writing – review & editing. CS: Investigation, Resources, Writing – review & editing. SL: Conceptualization, Data curation, Funding acquisition, Investigation, Methodology, Resources, Supervision, Writing – original draft, Writing – review & editing.
