# Supplementary material for: Toxoplasma gondii infection in people with schizophrenia is related to higher hair glucocorticoid levels
Source: Front Psychiatry. 2024 Feb 16;15:1286135. doi: 10.3389/fpsyt.2024.1286135 (PMC10904596; doi:10.3389/fpsyt.2024.1286135)
Supplement: Supplementary file 1 [file DataSheet_1.pdf]

Screening questionnaire (Phone interview)

Section 1 – Matching criteria

1. How old are you (in years)?

    ____

2. What gender are you?
    a. Female
    b. Male

3. What is your personal income in $ before income taxes for the past year.

    ____

    Refusal:

    If the participant refuse to answer question 3, go to question 3a)

    3 a) Is your personal income before income taxes for the past year
    a) Inferior to 20 000$ or
    b) Superior to 20 000$?

If the participant matches a table cell for which the number of participant is not yet attained, the questionnaire continue at section 2 (exclusion criteria). Otherwise the participant is excluded.

Table 1: Number of participant targeted by age, income and sex.

		Female	Male
18-29 years	< 20 000$	16	26
	> 20 000$	3	7
30-39 years	< 20 000$	12	19
	> 20 000$	7	11
40-59 years	< 20 000$	18	27
	> 20 000$	13	17
> 60 years	< 20 000$	9	6
	> 20 000$	3	5

Section 2 – Exclusion criteria

1. Have you ever been admitted in a psychiatric emergency in the last 5 years?
   a. Yes (Participant is excluded from study)
   b. No (Go to question 2)

2. Have you undergo an addiction treatment or visited a treatment centre in addiction related to your alcohol or drug use.
   a. Yes (Participant is excluded)
   b. No (Go to question 3)

3. (Women only) Are you presently pregnant or breastfeeding a baby?
   a. Yes (Participant is excluded)
   b. No (Go to question 4)

4. Do you take cortisone (cortisone is an anti-inflammatory medication) or a cortisone-based preparations?
   a. Yes (Participant is excluded)
   b. No (Go to section 3)

Section 3 – Assessment of psychotic symptoms.

Community Assessment of Psychic Experiences (CAPE)

a) For question 1 to 42,answers choices are :

☐ 1 - Never
☐ 2 - Sometimes
☐ 3 - Often
☐ 4 – Nearly always

If the participant answers “*never”*, go to next question, otherwise, check the choice that best reflects how you feel.

b) I am...

☐ 1 – Not distressed
☐ 2 – A bit distressed
☐ 3 – Quite distressed
☐ 4 – Very distressed

These questions refer to the past 12 months.

1. Do you ever feel as if people seem to drop hints about you or say things with a double meaning?

2. Do you ever feel as if some people are not what they seem to be?

3. Do you ever feel as if you are being persecuted in some way?

4. Do you ever feel as if there is a conspiracy against you?

5. Do you ever feel as if electrical devices such as computers can influence the way you think?

6. Do you ever feel that people look at you oddly because of your appearance?

7. Do you ever feel as if the thoughts in your head are being taken away from you?

8. Do you ever feel as if the thoughts in your head are not your own?

9. Have your thoughts ever been so vivid that you were worried other people would hear them?

10. Do you ever hear your own thoughts being echoed back to you?

11. Do you ever feel as if you are under the control of some force or power other than yourself?

12. Do you ever hear voices when you are alone?

13. Do you ever hear voices talking to each other when you are alone?

14. Do you ever feel as if a double has taken the place of a family member, friend or acquaintance?

15. Do you ever see objects, people or animals that other people cannot see?

Score of positive symptoms:

Frequency (part a):

Score A = Sum (A1 to A15) / 20

Distress (part b):

Score B = Sum (B1 to B15) / 20

Total (A & B)

Total score [1 - 8] = Score A + Score B

If total score ≥ 3.2, participant is excluded, else go to section 4.

Section 4: Assessment of bipolar disorder.

Mood disorder questionnaire (MDQ)

These questions refer to the past 12 months.

**1**	Has there ever been a period of time when you were not your usual self and...	**YES (1)**	**NO (0)**
**a**	...you felt so good or so hyper that other people thought you were not your normal self or you were so hyper that you got into trouble?		
**b**	...you were so irritable that you shouted at people or started fights or arguments?		
**c**	...you felt much more self-confident than usual?		
**d**	...you got much less sleep than usual and found you didn't really miss it?		
**e**	...you were much more talkative or spoke much faster than usual?		
**f**	...thoughts raced through your head or you couldn't slow your mind down?		
**g**	...you were so easily distracted by things around you that you had trouble concentrating or staying on track?		
**h**	...you had much more energy than usual?		
**i**	...you were much more active or did many more things than usual?		
**j**	...you were much more social or outgoing than usual, for example, you telephoned friends in the middle of the night?		
**k**	...you were much more interested in sex than usual?		
**l**	...you did things that were unusual for you or that other people might have thought were excessive, foolish, or risky?		
**m**	...spending money got you or your family into trouble?		
**2**	If you checked YES to more than one of the above, have several of these ever happened during the same period of time? ☐ YES (1) ☐ NO (0)		
**3**	How much of a problem did any of these cause you – like being unable to work; having family, money or legal troubles; getting into arguments or fights? *Please circle one response only*. ☐ No Problem (1) ☐ Minor Problem (2) ☐ Moderate Problem (3) ☐ Serious Problem (4)		

Score 1 = Sum (Question 1a – 1n)

If Score 1 ≥ 6 and Question 2 = yes and Question 3 ≥ 3, the participant is excluded, else the participant is included in the study.

End of Screening
